# P2X2 receptors in pyramidal neurons are critical for regulating vulnerability to chronic stress

**DOI:** 10.7150/thno.72144

**Published:** 2022-05-01

**Authors:** Xiao-Jing Kuang, Can-Yuan Zhang, Bing-Yi Yan, Wei-Zhong Cai, Cheng-Lin Lu, Li-Jia Xie, Shu-Ji Li, Peng-Li Kong, Jun Fan, Shu-Min Pan, Ting Guo, Xiong Cao

**Affiliations:** 1 Key Laboratory of Mental Health of the Ministry of Education, Guangdong-Hong Kong-Macao Greater Bay Area Center for Brain Science and Brain-Inspired Intelligence, Guangdong Province Key Laboratory of Psychiatric Disorders, Department of Neurobiology, School of Basic Medical Sciences, Southern Medical University, Guangzhou 510515, P. R. China.; 2 Microbiome Medicine Center, Department of Laboratory Medicine, Zhujiang Hospital, Southern Medical University, Guangzhou, Guangdong 510515, P. R. China.

**Keywords:** P2X2 receptors, stress vulnerability, mPFC, chronic stress, synapse

## Abstract

**Rationale:** Stress is a major risk factor for the development of depression. However, the underlying molecular mechanisms of stress vulnerability in depression are largely uncharacterized.

**Methods:** P2X2 receptors (a major receptor for gliotransmitter-ATP) in the medial prefrontal cortex (mPFC) were identified by real-time qPCR, western blots and RNAscope *in situ* hybridization in chronic social defeat stress model (CSDS). We generated P2X2 conditional knockout mice and overexpressed AAV-P2X2 in *CamkIIα-Cre* mice. The depression-like behaviors were assessed via CSDS, subthreshold social defeat stress (SSDS), social interaction test (SI), forced interaction test (FIT), forced swimming test (FST), sucrose preference test (SPT), novel stressed feeding (NSF) and open field test (OFT). The neuronal activity and synapse function of P2X2 receptors in the mPFC were detected by *in vivo* fiber-photometry, patch-clamp techniques and neuronal morphometric analysis.

**Results:** We identified that P2X2 receptors were increased in the mPFC of susceptible mice in CSDS. Conditional knockout of P2X2 receptors in pyramidal neurons promoted resilience of chronic stress-induced depressive-like behaviors, whereas pyramidal neurons - specific gain of P2X2 in the mPFC increased vulnerability to depressive-like behaviors. *In vivo* fiber-photometry, electrophysiology and neuronal morphometric analysis showed P2X2 receptors regulated neuronal activity and synapse function in the mPFC.

**Conclusions:** Overall, our studies reveal a critical role of P2X2 in mediating vulnerability to chronic stress and identify P2X2 as a potential therapeutic target for treatment of stress-related mood disorders.

## Introduction

Major depressive disorder (MDD) is a common and debilitating neurobiological illness that affects approximately 17% of the world's population in lifetime, and that is associated with high suicidal risk and serious economic burden 1-3. Given that stress has long-lasting adverse effects on brain function 4, 5, defects in the normal adaptive responses to stress can increase the likelihood of risk of developing depression 6. Stress exposure to mice develops social avoidance or anhedonia and is associated with increased vulnerability to stress. However, the mechanism of stress induced vulnerability of depression is largely unknown.

Adenosine triphosphate (ATP) is not only supported as energy storage within cells but is also a transmitter or cell-to-cell signaling molecule in central nervous systems 7, 8. ATP has been demonstrated to regulate a number of behaviors, involving mood and motivation, learning and memory, sleep and arousal, locomotor and feeding activities and cognition, via ligand-gated cationic channels (P2X receptors, P2XRs) or G protein-coupled receptors (P2YRs) 9-12. However, the dysregulation of ATP signaling was implicated in many psychiatric disorders, including depression, anxiety and posttraumatic stress disorder (PTSD) 7, 13, 14. Indeed, our previous study found that astrocyte-derived ATP modulates depressive-like behaviors 15, 16. P2XRs, expressed on neurons, regulate synaptic plasticity and coordinate synaptic networks 17-19. And P2X7R, expressed on microglia and macrophage, play a role in mediating stress and depression 20-23. However, whether and how neuronal P2XRs play a role in depression, especially in response to psychosocial stress, remains largely unexplored.

Here, we showed that P2X2 levels were increased in the mPFC of depression susceptible mice, and selective knockout and overexpression of P2X2 in mPFC pyramidal neurons bidirectionally regulated depressive-related behaviors. Furthermore, combining *in vivo* fiber-photometry, whole cell patch-clamp techniques and neuronal morphometric analysis, we present our study elucidated that P2X2 receptor in mPFC pyramidal neurons modulated vulnerability to chronic stress.

## Materials and Methods

### Mice

The mice were housed in groups of 4-6 per cage and were maintained under standard housing conditions in a temperature (24 ± 1 °C) controlled animal room on a 12 h light/dark cycle (lights were on from 7:00 A.M. to 7:00 P.M. every day) with food and water available *ad libitum*. Male C57BL/6J mice (aged 10-12 weeks) were obtained from Southern Medical University Animal Center (No: SCXK-2016-0041, Guangzhou, China) and allowed 1 week of acclimation to the housing facilities before the start of experiments.

#### *P2rx2-floxed* mice

This mouse line was generated by the Shanghai Biomodel Organism Science and Technology Development. Briefly, the floxed *P2rx2* allele was generated by introduction of *loxP* sites flanking the coding region of exon 8-11 of the *P2rx2* locus into the mouse genome. Recombinant embryonic stem cells were injected into C57BL/6J blastocysts to produce chimaeras which were then crossed to C57BL/6J mice for at least 10 generations to produce mice heterozygous for the floxed *P2rx2* allele.

#### *CamkIIα-Cre;P2rx2^loxp/loxp^* mice

The *CamkIIα-Cre* transgenic mice in a C57BL/6J genetic background were purchased from the Jackson Laboratory (Stock Number #005359, USA). *CamkIIα-Cre;P2rx2^loxp/loxp^* mice were generated by crossing *P2rx2^loxp/loxp^* mice with *CamkIIα-Cre* mice. Littermate *P2rx2^loxp/loxp^* mice were used as the controls.

#### *CamkIIα-Cre^ERT^;P2rx2^loxp/loxp^* mice

The *CamkIIα-Cre^ERT^* transgenic mice in a C57BL/6J genetic background were purchased from the Jackson Laboratory (Stock Number #012362, USA). *CamkIIα-Cre^ERT^;P2rx2^loxp/loxp^* mice were generated by crossing *P2rx2^loxp/loxp^* mice with *CamkIIα-Cre^ERT^* mice. To excise the *loxP* sites with *Cre* recombination, 2-month-old male mice were intraperitoneally injected with tamoxifen (TAM, Sigma-Aldrich, USA) once a day (100 mg per kg of body weight) for 5 days. TAM was made freshly by dissolving in a vehicle of corn oil (Sigma-Aldrich, USA) and ethanol solution (9 : 1 vol/vol) at a final concentration of 10 mg/ml. Littermate *P2rx2^loxp/loxp^* mice injected with TAM were used as the controls. Behavioral tests were conducted after 1 month.

All experiments were conducted in accordance with the Chinese Council on Animal Care Guidelines. Double-blinded behavioral tests were performed between 1:00 and 4:00 P.M., social interaction tests were performed between 6:00 P.M. and 11:00 P.M.

### RNA isolation and real-time quantitative PCR

Brain tissue samples were homogenized in RNAiso Plus (Takara, Japan) and processed according to the manufacturer's instructions. cDNA was synthesized from 500 ng of extracted total RNA using PrimeScript^TM^ RT Reagent Kit (Takara, Japan) according to the manufacturer's protocol. Quantitative RT-PCR was performed with SYBR-Green premix Ex Taq (Takara, Japan) and detected by a Real Time PCR System (ABI 7500, Thermo Fisher Scientific, USA). Following PCR amplification, a first derivative melting- curve analysis was performed to confirm the specificity of the PCR. Each reaction was performed in duplicate and analyzed according to the standard ΔΔC_t_ method using *β-actin* as a normalization control. The qPCR primers used in this study were listed in Table S1.

### Western blots analysis and quantification

Western blots were performed as described previously 24. The following primary antibodies were used: mouse anti-P2X2 monoclonal antibody (mAb) (1:500; Youke Biotech); rabbit anti-Flag mAb (1:1,000; Sigma-Aldrich, F7425); mouse anti-GAPDH mAb (1:5,000; Good Here, AB-P-R001); The polyvinylidene fluoride (PVDF) membranes were washed and incubated for 1 h at room temperature with the corresponding secondary antibodies: horseradish peroxidase (HRP)-conjugated goat anti-rabbit IgG (1:5,000; ZSGB-Bio); HRP-conjugated goat anti-mouse IgG (1:5,000; ZSGB-Bio). Peroxidase activity was detected with SuperSignal WestPico chemiluminescent substrate (Pierce Biotechnology) and visualized and digitized with BIO-RAD Gel Doc XR imaging system (BIO-RAD, Germany). Protein levels, quantified by computer analysis as the ratio between each immunoreactive band and the levels of GAPDH, were expressed as a fold change of vehicle-treated control.

### Immunofluorescence

Mice were deeply anesthetized with sodium pentobarbital and perfused with phosphate buffered saline (PBS) followed by 4% paraformaldehyde. Brains were dissected out, post-fixed, rinsed and immersed in 30% sucrose solution. Coronal sections (40 μm) containing mPFC or hippocampus were taken on a freezing microtome (CM1850, Leica, Germany). Free-floating sections were blocked in 10% normal goat serum within 0.3% triton X-100 for 2 h at room temperature and then incubated with one or two primary antibodies overnight at 4 °C, followed by washout and second antibodies incubation for 2 h at room temperature. Brain sections were mounted on superfrost slides, dried and coverslipped with Vectashield mounting medium containing 4,6-diamidino-2-phenylindole (DAPI, Vector Laboratories Inc., USA). Primary antibodies used included mouse anti-NeuN (1:300, Millipore, USA), mouse anti-GFAP (1:300, Cell Signaling, USA) and rabbit anti-Flag (1:500; Sigma-Aldrich, USA). Secondary antibodies used included Alexa Fluor 488 goat anti-mouse IgG, Alexa Fluor 594 goat anti-mouse IgG and Alexa Fluor 594 goat anti-rabbit IgG (Invitrogen, USA) were diluted 1:500. High-resolution images of regions of interest were acquired with an A1R confocal microscope (Nikon Instruments Inc., Japan). Images represent maximum intensity projections of 3-7 μm confocal stacks.

### RNAscope *in situ* hybridization

Mice were deeply anesthetized with sodium pentobarbital and perfused with PBS followed by 4% paraformaldehyde. Brains were dissected out, post-fixed, rinsed and gradually immersed in 10%, 20% and 30% sucrose solution. Every time we have to wait for the tissues to sink to the bottom of the container. Then the tissues were embedded with OCT, frozen with liquid nitrogen, and stored at -80 °C. Cryostat sections (10 μm) were collected and RNAscope hybridizations were carried out according to the manufacturer's instructions, using the RNAscope Multiplex Fluorescent Manual Assay kit (Advanced Cell Diagnostics, USA). Briefly, brain sections were dehydrated in sequential incubations with ethanol, followed by 30 min Protease III treatment and washing in 1× PBS. Appropriate combinations of hybridization probes were incubated for 2 h at 40 °C, followed by four amplification steps, DAPI counterstaining, and mounting on uperfrost Plus Adhesion Microscope Slides (Thermo Fisher Scientific, USA) mounting with Prolong Gold mounting medium (Thermo Fisher Scientific, USA). For each mouse, 4 bregma-matched sections were imaged. Images (2 per brain section) were acquired with Confocal Microscope with identical settings across control and *P2rx2*-cKO brain sections and represented as maximum intensity projections of acquired confocal z stacks. Dual-probe analysis was done with the CellProfiler software (v.3) 25, 26 with the following specifications for different target probes: probes against *P2rx2* (ACD no. 564511-C2) and *CamkIIα* (ACD no. 445231) were tested. Target probe (*P2rx2*) was labeled by fluorophore Opal 570, while cell-type marker (*CamkIIα*) was labeled by fluorophore Opal 520. Imaging analysis was performed with the following specifications for target probe (*P2rx2*): only puncta with a diameter between 6 and 15 pixels that were located within a pyramidal neurons' perinuclear space (within 70 pixels of the DAPI-positive nuclei) were quantified. Pyramidal neurons were defined as cells that contained at least two *CamkIIα*^+^ puncta (diameter 6-15 pixels).

### Cell culture

PFC tissues from 18-day-old C57BL/6J mouse embryos were isolated and maintained in ice-cold PBS for dissociation by using a pair of sterile operating scissors. Then they were incubated with 0.25% trypsin (Invitrogen) in 0.5 mM EDTA at 37 °C for 10 min. Culture medium was added and the cell suspension was transferred into 15 ml tubes and centrifuged at 900 g for 5 min. The pellet was resuspended in 10 ml of culture medium (Neurobasal Medium 500 ml + B27 10 ml + Glutamax-100X 5 ml). The neurons were placed in culture dishes and incubated in a humidified incubator containing O_2_/5% CO_2_/95% air at 37 °C. For the ATPᵞS experiments, the neurons were treated with ATPᵞS (50 μM/ml, Cat: A1388, Sigma-Aldrich) for 72 h. For the ATPase experiments, the neurons were treated with ATPase (30 units/ml, apyrase from potato, Cat: A7646, Sigma-Aldrich) for 24 h.

### Viruses

For P2X2 overexpression, the viruses AAV-Ef1a-DIO-P2rX2-3FLAG-WPRE (AAV2/9, 1.11 × 10^13^ vg/ml) and its control viruses pAOV-Ef1a-DIO-mCherry (AAV2/9, 2.02 × 10^13^ vg/ml) were produced by Shanghai Obio Technology Corp., Ltd. China. For sparse labeling, the viruses rAAV-Ef1a-DIO-FLP-WPRE-pA (AAV2/9, 2.48 × 10^12^ vg/ml) and rAAV-nEf1a-fDIO-EYFP-WPRE-pA (AAV2/9, 2.00 × 10^12^ vg/ml) were purchased from BrainVTA Co. China. For Ca^2+^ fiber photometry, the viruses rAAV-hSyn-GCaMP6s-WPRE-pA (AAV2/9, 5.40 × 10^12^ vg/ml) were purchased from BrainVTA Co. China. For labelling and recording pyramidal neurons in mPFC in whole cell patch-clamp, AAV-mCamkIIα-EGFP-WPRE-pA (AAV2/9, 1.77 × 10^13^ vg/ml) were purchased from Taitool Bioscience, China. For mPFC local P2X2 receptors knockdown experiment, the viruses AAV-mCamkIIα-H2B-EGFP-P2A-iCre-WPRE-pA (AAV2/9, 1.35 × 10^13^ vg/ml) and AAV-mCamkIIα-EGFP-WPRE-pA (AAV2/9, 1.77 × 10^13^ vg/ml) were purchased from Taitool Bioscience, China.

For sparse labeling, virus application is depending on FLP/FRT-mediated site-specific recombination system. Using this system, open reading frame (fDIO) that expressed EYFP was double-FRT-flanked so that the EYFP expression is Flp-dependent. Thus, we can achieve the sparse labeling purpose by applying low titer AAV-DIO-Flp virus.

### Stereotactic injection

Stereotactic surgery was performed on mice under sodium pentobarbital anesthesia and positioned in a small animal stereotaxic instrument (RWD life Science, China). The skull surface was exposed and 33-gauge syringe needles (Hamilton Co.) were used to bilaterally injected AAVs (0.5 μl for each site) into mPFC at a rate of 0.1 μl per min with the following coordinates: 1.9 mm anterior-posterior (A/P), 0.35 mm medial-lateral (M/L), 2.7 mm dorsal-ventral (D/V) from bregma. 0.5 μl of virus was injected over 5 min. Following a 5 min delay, the needle was pulled up 0.3 μm, and an additional 0.5 μl of the virus was injected. After surgical procedures, mice were returned to their home cage at least 21 days to allow for maximal gene expression.

### Optical fiber implantation

Ten minutes after virus injection, a ceramic ferrule with an optical fiber (for fiber photometry: 200 μm in diameter, NA of 0.37) was implanted with the fiber tip above the mPFC using a stereotaxic instrument (RWD life Science, China). Then the ferrule was secured onto the skull with dental adhesive cement. The fiber-photometry experiments were conducted 2 weeks after optical fiber implantation.

### Behaviors

#### Chronic social defeat stress (CSDS)

CSDS was performed as previously described 27. Briefly, experimental mice were subjected to physical interactions with an unfamiliar CD-1 mouse for 10 min once per day over 10 consecutive days. Following the daily defeat, the experimental mouse and the aggressor were separated by a perforated translucent plastic divider, allowing sensory contact, over the subsequent 24 h period. Unstressed control mice were housed two per cage in either side of a perforated divider and rotated daily in a similar manner without being exposed to the CD-1 mice. The social interaction (SI) test was conducted 24 h after the last defeat.

#### Subthreshold social defeat stress (SSDS)

A subthreshold variation of the CSDS protocol was used to evaluate increased susceptibility to stress 28. Experimental mice were subjected to physical interactions with an unfamiliar CD-1 for 10 min once per day over 3 consecutive days. Following the daily defeat, the experimental mouse and the aggressor were separated by a perforated translucent plastic divider, allowing sensory contact, over the subsequent 24 h period. The SI test was conducted 24 h later.

#### Social interaction test (SI)

SI test was performed as previously described under red-light conditions 27. The procedure consisted of two phases of 2.5 min each. First, mice were allowed to explore freely in the first 2.5 min (no target) in a Plexiglas open-field arena (42 cm × 42 cm × 42 cm, Nationwide Plastics) with a small animal cage placed at one side of the arena. At the end of 2.5 min, the mouse was removed and the arena was cleaned. Then, during the second 2.5 min, the mouse was reintroduced back into the arena in the presence of a novel caged CD1 mouse inside the small animal cage (with target). Movements were monitored and recorded automatically with a tracking system (Ethovision 7.0, Noldus Information Technology, Netherlands) to determine exploratory behavior and locomotion. SI ratio was calculated by dividing the time spent in the interaction zone when the CD1 mouse was present divided by the time spent in the interaction zone when the CD1 mouse was absent. All mice with a SI ratio below 1.0 were classified as susceptible (sus.) and all mice with a SI ratio above 1.0 were considered as resilient (res.).

#### *In vivo* fiber-photometry

*In vivo* fiber-photometry was performed as previously described 29, 30. We measured bulk fluorescence from mPFC using a single optical fiber for both delivery of laser and collection of emitted fluorescence. The fluorescence output of GCaMP6s is modulated by varying the intensity of the laser, generating an amplitude-adjusted fluorescence signal that is demodulated to recover the original response. A laser beam from a laser tube (488 nm) was reflected by a dichroic mirror focused by a 10× (NA of 0.3) lens and coupled to an optical commutator. The fluorescence was bandpass filtered (MF525-39, Thorlabs) and collected by a photomultiplier tube (R3896, Hamamatsu). An amplifier (C7319, Hamamatsu) was used to transform the photomultiplier tube current output to voltage signals, which were further filtered through a low-pass filter (40 Hz cutoff; Brownlee 440). In order to minimize photobleaching, the laser power at the fiber tip was adjusted to 30 μW.

All the neuronal calcium signals were recorded during the forced interaction test. The optical fiber tip sites were histologically examined in each mouse after finished the experiments. Bulk fluorescence signals were generated and analyzed with MATLAB programs. The fluorescence responses for a population of neurons were calculated using the following formula: Z-score = (F_Signal_ - F_Basal_) / STD (F_Basal_). F_Basal_**:** average value of F_Basal_ in baseline time; STD (F_Basal_): standard deviation of F_Basal_ in baseline time.

#### Forced interaction test (FIT)

The FIT experiment was carried out simultaneously with *in vivo* optical fiber calcium signal recording and was performed as previously described under red-light conditions 29. The detection process consists of two stages, each of which takes 5 min. Firstly, in the first 5 min (without CD1 mouse), the experimental mice were put into a wire mesh box in the center of a new detection chamber and allowed to move freely; after 5 min, a strange CD1 mouse (target) was introduced into the detection chamber, and the CD1 mouse could move freely in the detection chamber, and randomly attacked the experimental mice in the wire mesh box for 5 min. However, CD1 mouse could not touch or bite the mice directly. When the CD1 mouse touches or reaches into the barbed wire, it is regarded as an effective attack. The changes of Ca^2+^ signal activity of pyramidal neurons in mPFC were observed by optical recording system at the moment of attacked by CD1 mice. The mice were put into the test room one hour in advance for adaptation.

#### Forced swimming test (FST)

Mice were gently placed in a transparent glass cylinder (height 45 cm, diameter 19 cm) of water (22 °C -25 °C, 23 cm in height) under bright light conditions and videotaped for 6 min. Movements were monitored and recorded automatically with a tracking system (Ethovision 7.0, Noldus Information Technology, Netherlands). The immobility time (5% threshold) was automatically analyzed during the last 4 min of a 6 min test session. Immobility was defined as no movement at all or only minor movements necessary to keep the nose above the water versus mobility, which was defined by swimming and struggling behaviors.

#### Sucrose preference test (SPT)

Depression-associated behaviors were assessed by measuring sucrose preference. Briefly, mice were singly caged for 24 h habituation before test, and had their normal water bottle removed and replaced with two 50-ml bottles (A and B) filled with water. On the test days, water from bottle A was replaced with 1% sucrose solution, and bottle B contained water. All bottles were weighed, and mice were allowed 24 h to drink. Then bottles were reweighed, and to prevent possible effects of side-preference in drinking behavior, the position of the bottles in the cage were switched before a second 24 h period of drinking. At the end of the second day, sucrose preference was calculated as the total amount of sucrose consumption divided by the total amount of fluid consumed over the two days of sucrose availability. No previous food or water deprivation was applied before the test.

#### Novelty-suppressed feeding test (NSF)

After 24 h of food deprivation and water available *ad libitum*, mice were placed in a brightly lit open arena (50 × 50 × 50 cm) containing clean wood chip bedding. A round filter paper (8 cm diameter) was placed in the center of the arena, and one familiar food pellet was placed on the center of the filter paper. Mice were removed from their home cage, placed in a holding cage for 60 min before testing and then placed in a corner of the testing arena. The latency to begin a feeding episode was recorded with a video camera suspended above the arena and saved for further analysis (Ethovision 7.0, Noldus Information Technology, Netherlands). Immediately after testing, mice were removed from the arena and placed into their home cage to measure food consumption for 5 min.

#### Open field test (OFT)

Mice were placed in an open chamber (Accuscan Instruments, USA) with transparent, plastic walls and locomotion was monitored and tracked for 5 min. The total distance traveled across a session was analyzed using Versmax analyzer software.

### Whole-cell patch-clamp recording

In order to specifically label and record pyramidal neurons, the mice were injected with AAV-CamkIIα-EGFP in mPFC. Slices were prepared as previously described 31. Male mice were anesthetized with diethylether and then decapitated. The brains were removed quickly and placed into ice-cold modified artificial cerebrospinal fluid (ACSF) containing (in mM) 250 sucrose, 26 NaHCO_3_, 10 glucose, 10 MgSO_4_, 2 KCl, 1.3 NaH_2_PO_4_ and 0.2 CaCl_2_. Slices containing the mPFC (300 µm) were prepared in ice-cold modified ACSF using a VT-1200S vibratome (Leica, Germany), transferred to the storage chamber containing regular ACSF (in mM) (126 NaCl, 26 NaHCO_3_, 10 glucose, 3 KCl, 2 CaCl_2_, 1.25 NaH_2_PO_4_ and 1 MgSO_4_), and allowed to recover at 34 °C for 30 min and then at room temperature for one hour before recording. During the slice preparation, all solutions were saturated with 95% O_2_/5% CO_2_.

Slices were placed in the recording chamber that was superfused (3 ml/min) with ACSF between 30-32 °C. Whole-cell patch-clamp recordings of mPFC pyramidal neurons were obtained under an infrared (IR)-differential interference contrast (DIC) microscope (ECLIPSE FN1, Nikon). To record mEPSCs, pipettes (input resistance:3-7 MΩ) were filled with an intracellular solution containing (in mM): 140 K-gluconate, 9 HEPES, 4.4 Phosphocreatine disodium, 4 ATP-Mg, 4.5 MgCl_2_, 0.3 GTP, and 5 EGTA, at 290-300 mOsm, pH 7.2-7.3, adjusted with KOH. When mEPSCs were recording, the GABA_A_ receptors and action potentials were blocked with 20 µM bicucullinemethiodide (BMI) and 1 µM tetrodotoxin (TTX), respectively. Recordings were discarded if series resistances changed by >20%. Measurements of membrane potentials were not corrected for the liquid junction potential error. Data were acquired with an EPC 10 amplifier (HEKA Elektronik), filtered at 2.9 kHz with a Bessel filter, digitized at 10 kHz, and analyzed using pClamp10.2 software (Molecular Devices).

### Morphometric analysis

For three-dimensional Sholl analysis, total dendritic length and spine morphology were calculated by using software Imaris 8.1. Briefly, a z-stack acquisition was imported, calibrated in Imaris, and manually traced. Total dendritic length was then computed. For Sholl analysis, the shell interval was set at 10 μm. All analyses were performed blindly.

### Statistics

All statistical analyses were performed using SPSS 20. software. Results were expressed as mean ± SEM. Statistical significance was calculated by unpaired two-tailed Student's t-test, one-way ANOVA with Bonferroni's *post hoc* tests or two-way ANOVA with Bonferroni's *post hoc* tests based on the design of experiment. P < 0.05 was considered significant. For each experiment, the tests used, as well as the main effect and *post hoc* statistical significances, are as given in the appropriate figure legend.

## Results

### Social stress increases neuronal P2X2 expression in the mPFC

**Figure 1:**
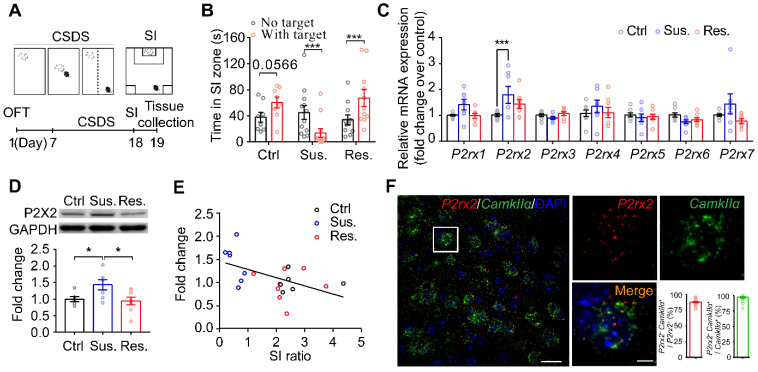
** Social stress increases neuronal P2X2 expression in the mPFC.** (**A**) Schematic of the CSDS paradigm. (**B**) Statistical comparison of time in SI zone, SI: social interaction, Ctrl: control, Sus.: susceptible, Res.: resilient (ctrl: t_(14)_ = 1.941 p = 0.057, Sus.: t_(20)_ = 2.276, p = 0.030, Res.: t_(22)_ = 2.514, p = 0.020; multiple t test). (**C**) mRNA levels of gene profiles related to P2X receptors in the mPFC of Sus, Res. and Ctrl mice following chronic social defeat stress (CSDS) paradigm (n = 7-8, one-way ANOVA). (**D**) Western blots representation (top) and quantification (bottom) of P2X2 protein level in the mPFC after CSDS (n = 5-7, p = 0.022, one-way ANOVA). (**E**) Pearson's correlation analyses of P2X2 protein level and social avoidance, Pearson's squared correlation coefficient (R^2^) and p value are shown at the bottom right of the plot (n = 5-7). (**F**) Double-fluorescent RNAscope in situ hybridization probing *CamkIIα* (green) and *P2rx2* (red) in the mPFC of adult C57BL/6J mice. Left, scale bar, 25μm; right, magnified view of left image, scale bars, 5 µm. The data are shown as mean ± SEM. *p < 0.05, **p < 0.01.

Chronic social defeat stress (CSDS) is a well-established model of depression that mimicked several psychopathological dimensions of depression 15, 27, 32, 33. The adult male C57BL/6J mice that have been exposed to CSDS could be divided into susceptible and resilient subpopulations by social interaction (SI) ratio (**Figure 1A-B, Figure S1**). To investigate the role of the P2XRs in depression, we firstly detected mRNA level of all P2XRs in the mPFC, a key brain region for pathogenesis of depression 34, 35, from the C57BL/6J mice after CSDS paradigm. Real-time qPCR analysis revealed significantly enhanced *P2rx2* mRNA expression in mPFC tissues from susceptible but not resilient mice compared with control mice (**Figure 1C**). Meanwhile, other P2XRs showed no obvious difference. Consequently, western blots analysis confirmed the increased P2X2 protein level in the mPFC of susceptible mice (**Figure 1D**). We further performed the correlation analyses between P2X2 protein level and behavior performance in the same individual animals. Interestingly, the P2X2 protein level in susceptible mice was negatively correlated with SI ratio, but not in resilient mice (**Figure 1E**). Previous works showed astrocytic ATP release was decreased in the mPFC in the CSDS model 15, 36, 37, and ATP regulated the dynamic change of the P2X2R 17, 38, 39. We found that P2X2 mRNA and protein levels were decreased after 72 h ATPᵞS treatment and increased after 24 h ATPase treatment in cultured neurons (**Figure S2**). These results suggested that the expression of P2X2 receptors varied inversely with the change of ATP concentration.

Stress induces mPFC pyramidal neuronal atrophy and loss in depression 40-42; and pyramidal neurons are predominate neurons in the mPFC, gating depressive-like behavior 43-45. We next quantified *P2rx2* levels in *CamkIIα*-positive neurons using RNAscope *in situ* hybridization. Quantitative analysis displayed that *P2rx2* mRNA were majorly presented in *CamkIIα*-positive neurons (**Figure 1F**). These data suggested that P2X2 in pyramidal neurons in mPFC is involved in depressive-like behaviors.

### The lack of P2X2 promotes resilience to stress-induced depressive-like behaviors

**Figure 2:**
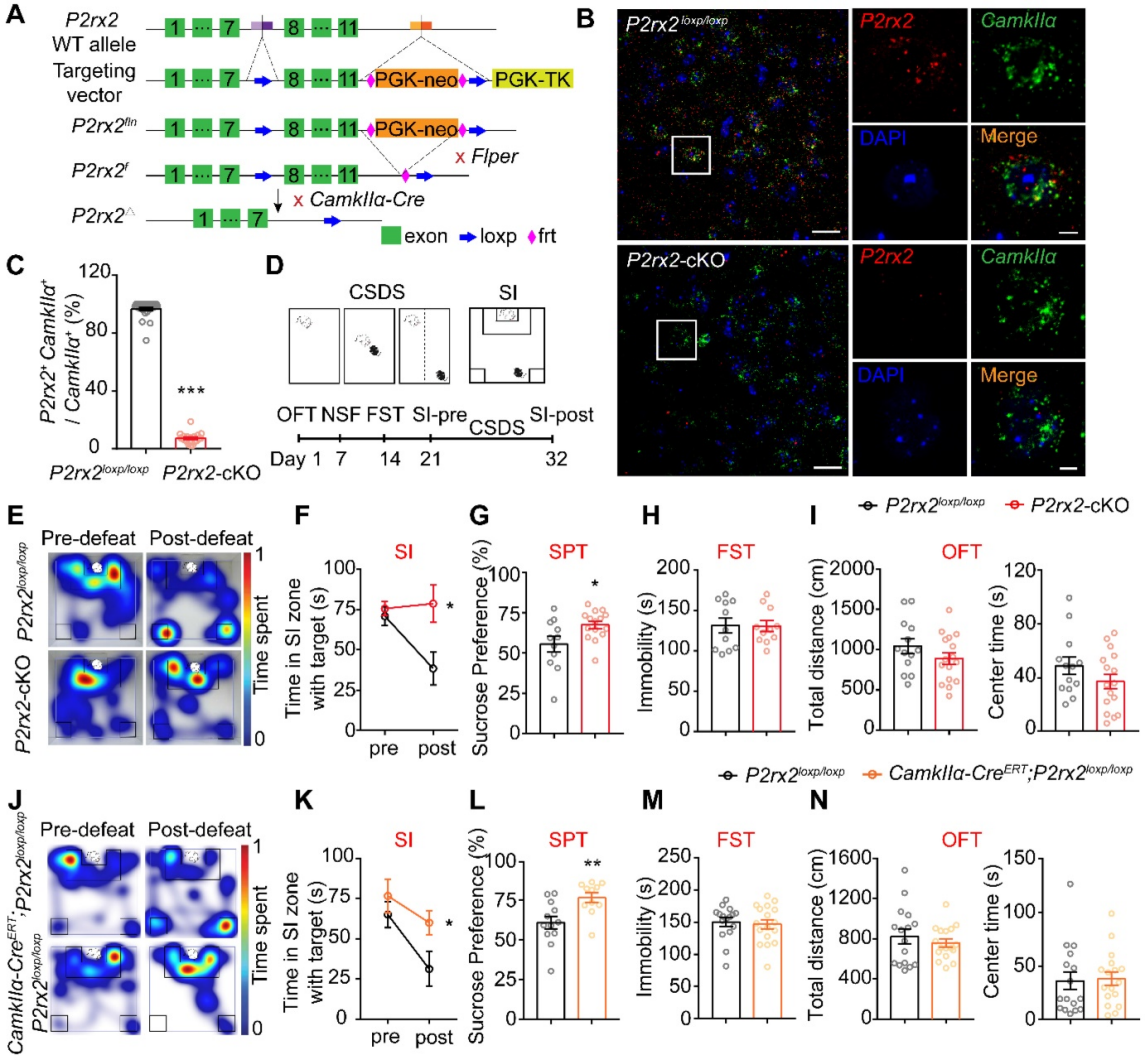
**The lack of *P2rx2* promotes resilience to stress-induced depressive-like behaviors.** (**A**) Gene targeting strategy for the generation of floxed *P2rx2* mice. In the targeting vector, a neomycin resistance cassette, FRT (open diamond) and two* loxp* (arrow) sites are shown. PGK-TK was used for negative selection. (**B-C**) RNAscope *in situ* hybridization micrographs (B) and enumeration analysis (C) (n = 19-28 views from 4-5 mice, t_(45)_ = 62.426, p < 0.001, unpaired t test) of *P2rx2*-cKO and control mice. *CamkIIα* (green), *P2rx2* (red). Left, scale bar, 25μm; right, magnified view of left image, scale bars, 5 µm. (**D**) Experimental timeline of behavioral studies. (**E**) Representative heatmaps of normalized time spent during SI test before (pre-defeat) and after (post-defeat) CSDS of *P2rx2*-cKO (bottom) and littermate control (top) mice. (**F-I**) Statistics analysis of *P2rx2*-cKO and control mice in SI test before and after CSDS (F) (n = 12-14, p = 0.049, interaction effect, matching two-way ANOVA), SPT (G) (n = 11-15, t_(24)_ = 2.418, p = 0.023, unpaired t test), FST (H) (n = 11 per group, t_(20)_ = 0.079, p = 0.940; unpaired t test), and total distance (t_(27)_ = 1.334, p = 0.190), center time (t_(27)_ = 1.387, p = 0.180) in OFT (I) (n = 13-16, unpaired t test). (**J**) Representative heatmaps of normalized time spent during SI test before (pre-defeat) and after (post-defeat) CSDS of *CamkIIα-Cre^ERT^; P2rx2^loxp/loxp^* (bottom) and littermate control (top) mice. (**K-N**) Statistics analysis of *CamkIIα-Cre^ERT^; P2rx2^loxp/loxp^* and littermate control mice in SI test before and after CSDS (K) (n = 8-13, p = 0.032, interaction effect, matching two-way ANOVA), SPT (L) (n = 11-12, t_(21)_ = 3.066, p = 0.006, unpaired t test), FST (M) (n=16-17, t_(31)_ = 0.348, p = 0.730; unpaired t test), total distance (t_(31)_ = 0.7819, p = 0.44), and center time (t_(31)_ = 0.193, p = 0.850) in OFT (N) (n = 16-17, unpaired t test). The data are shown as mean ± SEM. *p < 0.05, **p < 0.01.

To detect whether P2X2 in pyramidal neurons plays a role in depressant-related behaviors, we crossed mice with *P2rx2* allele in which *loxp* sites flank exon 8-11 with *CamkIIα-Cre* mouse to generate pyramidal neuron *P2rx2* conditional knockout mice: *CamkIIα-Cre;P2rx2^loxp/loxp^* (*P2rx2*-cKO) (**Figure 2A**). *P2rx2*-cKO mice grew well to normal size, and deletion of *P2rx2* did not lead to gross anatomical changes of brain or the densities of neurons and astrocytes (**Figure S3**). Next, to examine efficiency of *P2rx2* deletion in pyramidal neurons, we quantified *P2rx2* mRNA in *CamKIIα-*positive neurons in *P2rx2*-cKO and control mice using RNAscope *in situ* hybridization (**Figure 2B**). *P2rx2* mRNA expression in pyramidal neurons significantly decreased in mPFC of *P2rx2*-cKO mice compared with littermate control (**Figure 2C**), showed that the *P2rx2* was successfully knocked out in pyramidal neurons.

Then, we investigated the behavioral responses of *P2rx2*-cKO mice and littermate controls (**Figure 2D**). In CSDS paradigm, *P2rx2*-cKO and control mice showed no difference in social interaction under baseline, pre-defeat conditions (**Figure 2E-F, Figure S4A**). However, after 10 consecutive days social defeat stress, control mice decreased interaction time in SI test, whereas* P2rx2*-cKO mice blocked the development of such social avoidance (**Figure 2E-F, Figure S4A**). Moreover, *P2rx2*-cKO mice showed notably sucrose preference and indicated resistance to anhedonia phenotype in the sucrose preference test (SPT) (**Figure 2G**). And no behavioral differences were observed between *P2rx2*-cKO and littermate control mice in forced swimming test (FST) (**Figure 2H**), novelty-suppressed feeding test (NSF) (**Figure S4B**) and open filed test (OFT) (**Figure 2I, Figure S4C**).

In order to exclude the effects of P2X2 on embryonic development, we generated *CamkIIα-Cre^ERT^;P2rx2^loxp/loxp^* mice, in which *P2rx2* was specifically deleted in pyramidal neurons via Tamoxifen-inducible *Cre* recombination driven by the *CamkIIα* promoter. In the CSDS experiments, *CamkIIα-Cre^ERT^;P2rx2^loxp/loxp^* mice showed no behavioral deficit, while littermate control mice developed social avoidance (**Figure 2J-K, Figure S4D**). Furthermore, *CamkIIα-Cre^ERT^;P2rx2^loxp/loxp^* mice showed resistance to anhedonia as assessed with the SPT, which was consistent with the result of *P2rx2*-cKO mice (**Figure 2L**). And no differences were observed between *CamkIIα-Cre^ERT^; P2rx2^loxp/loxp^* and littermate control mice in FST (**Figure 2M**), NSF (**Figure S4E**) and OFT (**Figure 2N, Figure S4F**).

Taken together, these data indicated that selectively knockout *P2rx2* in pyramidal neurons promotes resilience to social defeat-induced social avoidance and anhedonia.

### Loss- or gain-of-function of P2X2 in mPFC pyramidal neurons bidirectionally regulates susceptibility to stress

**Figure 3:**
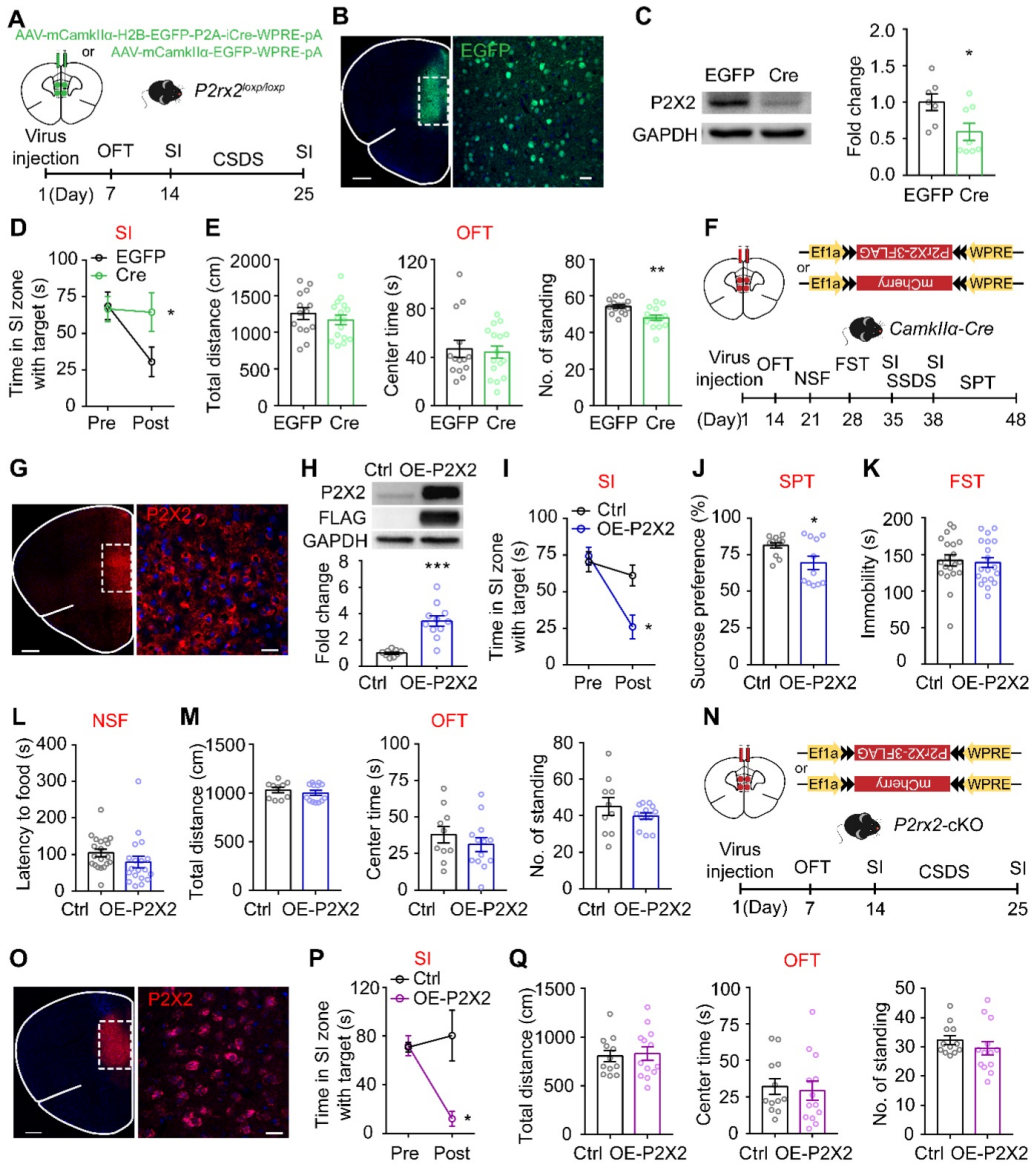
**P2X2 overexpression in mPFC enhances susceptibility to stress.** (**A, F, N**) Schematic of the experimental paradigm. (**B**) The specific expression of AAV-CamkIlα*-*Cre-EGFP (green) in mPFC of *P2rx2^loxp/loxp^* mice. Scale bar, 500 µm, zoom image, 25 µm. (**C**) Western blots and quantification of P2X2 protein levels in P2X2 knockdown and control mice (n = 9-11, t_(18)_ = 5.542, p < 0.001, unpaired t test). (**D-E**) Statistics analysis of mPFC P2X2 knockdown or control mice in SI test (D) (n = 10-11, p = 0.045, interaction effect, matching two-way ANOVA), and total distance (t_(28)_ = 0.845, p = 0.405), center time (t_(28)_ = 0.311, p = 0.758), number of standing (t_(28)_ = 3.569, p = 0.001) in OFT (E) (n = 14-16, unpaired t test). (**G, O**) The specific expression of P2X2 (red) in mPFC of *CamkIIα-Cre* (B) and *P2rx2*-cKO (J) mice. Scale bar, 500 µm, zoom images, 25 µm. (**H**) Western blots and quantification of P2X2 protein levels in overexpression and control mice (n = 9-11, t_(18)_ = 9.758, p < 0.001, unpaired t test). (**I-M**) Statistics analysis of *CamkIIα-Cre* mice that injected with AAV-DIO-P2rx2 or AAV-DIO-mCherry in SI test (I) (n = 7-9, p = 0.022, interaction effect, matching two-way ANOVA), SPT (J) (n = 11-12, t_(21)_ = 2.407, p = 0.025, unpaired t test), FST (K) (n=16-17, t_(31)_ = 0.348, p = 0.730; unpaired t test), NSF (L) (n = 11-12, t_(21)_ = 1.879, p = 0.070; unpaired t test), and total distance (t_(21)_ = 0.807, p = 0.430), center time (t_(21)_ = 0.907, p = 0.370), number of standing (t_(21)_ = 0.799, p = 0.290) in OFT (M) (n = 10-13, unpaired t test). (**P-Q**) Statistics analysis of *P2rx2*-cKO mice that injected with AAV-DIO-P2rx2 or AAV-DIO-mCherry in SI test (P) (n = 6-7, p = 0.011, interaction effect, matching two-way ANOVA), total distance (t_(23)_ = 0.239, p = 0.790), center time (t_(23)_ = 0.346, p = 0.730), number of standing (t_(23)_ = 1.011, p = 0.320) in OFT (Q) (n = 12-13, unpaired t test). The data are shown as mean ± SEM. *p < 0.05, **p < 0.01.

To determine whether the selective knockout of P2X2 receptors in the mPFC pyramidal neurons would induce antidepressant-like behaviors, we bilaterally injected AAV-CamkIIα-EGFP-Cre or AAV-CamkIIα-EGFP virus into the mPFC of *P2rx2^loxp/loxp^* mice for behavioral studies (**Figure 3A**). Confocal images showed AAV-CamkIIα-EGFP-Cre virus were correctly expressed in the mPFC pyramidal neurons of *P2rx2^loxp/loxp^* mice (**Figure 3B**). Western blots analysis showed that P2X2 protein level was decreased in local knockout mice (**Figure 3C**). After CSDS paradigm, the mice injected with control virus in the mPFC decreased interaction time in SI test, whereas the local P2X2 receptor knockout mice blocked the development of such social avoidance (**Figure 3D**, **Figure S5A-B**). And no obviously behavioral differences were observed in OFT (**Figure 3E**). This data was in accordance with performance of *P2rx2*-cKO mice.

We next tested whether P2X2 overexpression (OE-P2X2) in mPFC pyramidal neurons is sufficient to induce depressive-like behaviors by using a viral expression approach (**Figure 3F**). Confocal images showed AAV-DIO-P2RX2 virus was expressed in the mPFC neurons of *CamkIIα-Cre* mice (**Figure 3G**). Western blots analysis of mPFC confirmed the expression of P2X2-flag in mPFC (**Figure 3H**). For behavioral test, we adopted a three-day sub-threshold social defeat stress (SSDS) paradigm. After SSDS, OE-P2X2 mice spent less time in interaction zone with target during SI test, whereas control group showed no behavioral difference (**Figure 3I, Figure S5C-D**). Moreover, establishment of depressive-like behavior was confirmed in OE-P2X2 mice with SPT (**Figure 3J**). There was no effect in FST (**Figure 3K**), NSF (**Figure 3L, Figure S5E**) and OFT (**Figure 3M**).

To further assess whether the enhancement of P2X2 in mPFC pyramidal neurons is essential for the depressive-like effect, we injected AAV-DIO-*P2rx2* or control virus into the mPFC of *P2rx2*-cKO mice (**Figure 3N-O**). As expected, after 10 days CSDS, overexpression of P2X2 in the mPFC of *P2rx2*-cKO mice increased stress-induced social avoidance, whereas control virus infusion had no effect on resilience to depressant-like behavior of *P2rx2*-cKO mice (**Figure 3P, Figure S5F-G**). And no difference was observed in OFT (**Figure 3Q**).

Together, these results indicated that P2X2 in mPFC pyramidal neurons bidirectionally regulates stress-induced depressive-like behaviors.

### Conditional knockout or overexpression of P2X2 bidirectionally regulates neuronal Ca^2+^ activity under social stress

**Figure 4:**
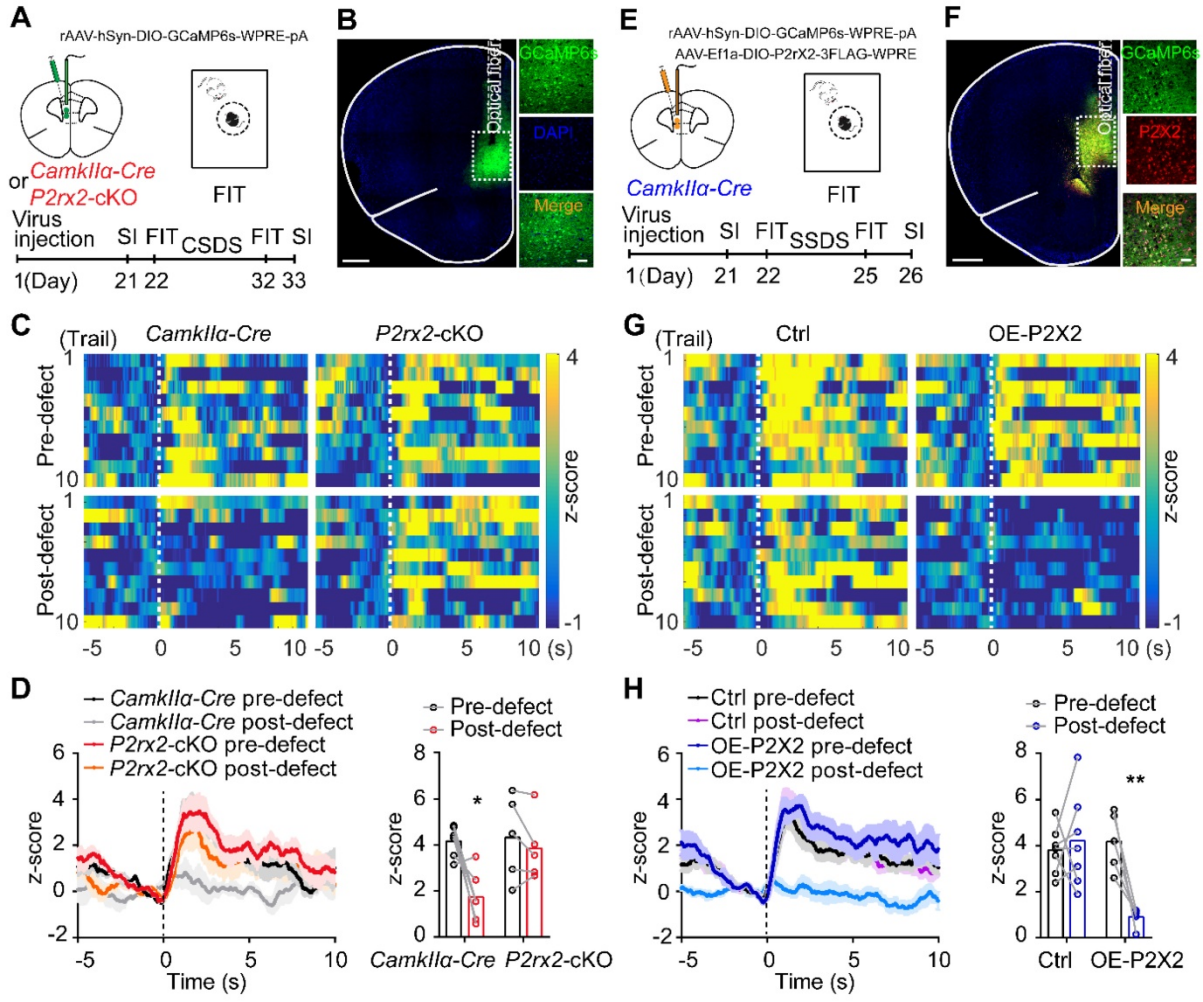
** Conditional knockout or overexpression of P2X2 bidirectionally regulates neuronal Ca^2+^ activity under social stress.** (**A, E**) Optic fiber placement, virus and experimental timeline used for fiber photometry. (**B, F**) Representative images of optical fiber track above the GCaMP6s (green) positive neurons in *P2rx2*-cKO (B) or P2X2 overexpression (P2X2, red) (F) mice. Scale bar, 500 µm, zoom images, 50 µm. (**C-D**) Representative heatmaps (C), average (D, left) and peak (D, right) z-score changes (n = 5-6, p = 0.050, interaction effect, matching two-way ANOVA) in normalized Ca^2+^ activity of *P2rx2*-cKO and control mice during FIT behavior. (**G-H**) Representative heatmaps (G), average (H, left) and peak (H, right) z-score changes (n = 6-7, p = 0.003, interaction effect, matching two-way ANOVA) in normalized Ca^2+^ activity of P2X2 overexpression and control mice during FIT behavior. The data are shown as mean ± SEM. *p < 0.05, **p < 0.01.

Multiple lines of evidence implicated that P2X2 can affect neuronal excitability and synaptic plasticity 18, 46. Thus, we examined whether P2X2 regulating depressive-related behaviors via mediating excitability of pyramidal neurons in mPFC, by using *in vivo* fiber photometry. In order to do this, we injected AAV-DIO-GCaMP6s into the mPFC of *P2rx2*-cKO or control mice and planted a fiber above the infected cells (**Figure 4A-B**). Before and after CSDS paradigm, we compared the GCaMP signals of pyramidal neurons in mPFC while mice were attacked by an aggressive CD1 mouse. On the day before CSDS, attack bouts elicited strong excitatory responses in both *P2rx2*-cKO and littermate control mice (**Figure 4C-D**). However, after 10 days CSDS, the robust excitatory responses still remained in *P2rx2*-cKO mice while no obvious responses were exhibited in control mice (**Figure 4C-D**). Interestingly, these Ca^2+^ responses were consistent with behavioral performances of *P2rx2*-cKO and control mice (**Figure S5A-B**). These results indicated that P2X2 conditional knockout sustained strong Ca^2+^ activity in mPFC pyramidal neurons in social stress.

Furthermore, we performed the similar *in vivo* fiber photometry of Ca^2+^ signals in OE-P2X2 and control mice (**Figure 4E-F**). On the day before SSDS, no significant change was observed between OE-P2X2 and control mice. After 3 days SSDS, P2X2 overexpression group showed notably decreased Ca^2+^ signals responses by compared with littermate control group (**Figure 4G-H**). These responses were consistent with behavioral performances of OE-P2X2 and control mice (**Figure S5C-D**). These data suggested that P2X2 overexpression decreased Ca^2+^ activity in mPFC pyramidal neurons under social stress.

### P2X2 modulates excitatory synaptic transmission in mPFC under social stress

**Figure 5:**
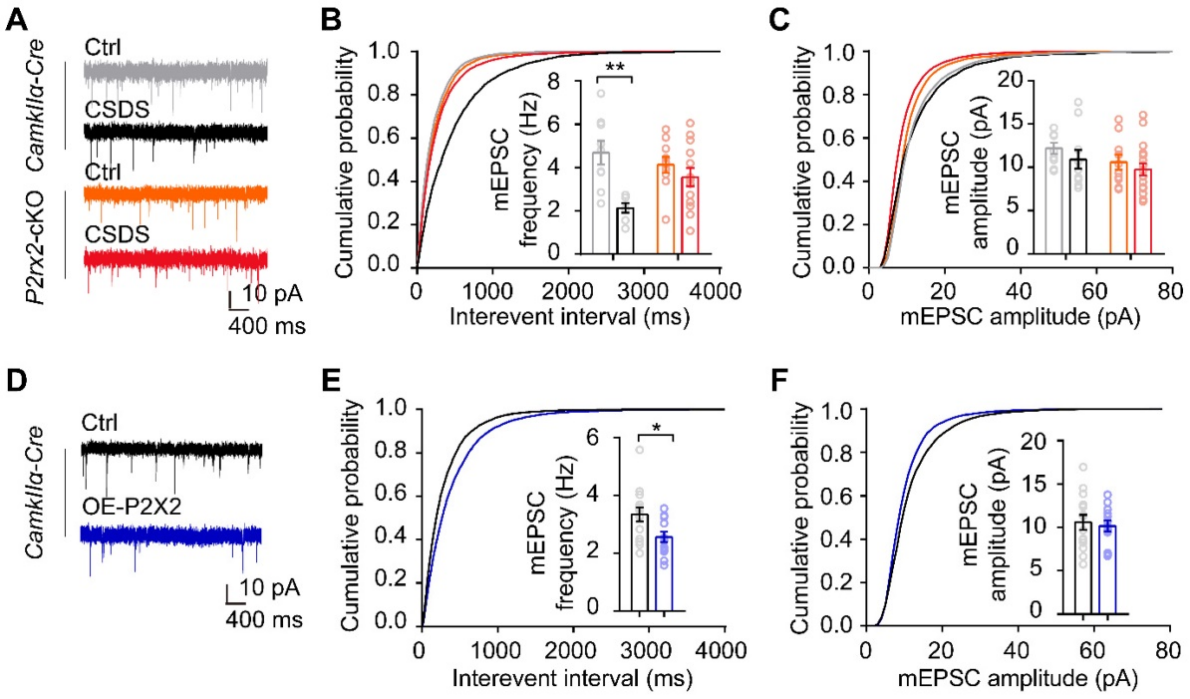
** P2X2 modulates excitatory synaptic transmission in mPFC under social stress.** (**A-C**) Representative traces of mEPSCs (A), cumulative distributions and average frequencies of mEPSCs (B) (n = 7-13 neurons from 3-5 mice, p = 0.030, interaction effect, two-way ANOVA) and cumulative distributions and average amplitudes of mEPSCs (C) (n = 7-13 neurons from 3-5 mice, p = 0.550, interaction effect, two-way ANOVA) in the mPFC of *P2rx2*-cKO mice or control littermates after CSDS paradigm. (**D-F**) Representative mEPSCs recordings (D), cumulative distributions and average frequencies of mEPSCs (E) (n = 13-14 neurons from 3 mice/group, t_(25)_ = 2.520, p = 0.019, unpaired t test), cumulative distributions and average amplitudes of mEPSCs (F) (n = 13-14 neurons from 3 mice/group, t_(25)_ = 0.398, p = 0.694, unpaired t test) in the mPFC of P2X2 overexpression or control mice. The data are shown as mean ± SEM. *p < 0.05, **p < 0.01.

To investigate whether the change of P2X2 level is crucial to synaptic functions under stress, we performed whole-cell patch-clamp recordings in acute mPFC slices of *P2rx2*-cKO mice and littermate control. Recordings and analysis from pyramidal neurons of control mice showed significantly lower frequencies of miniature excitatory postsynaptic currents (mEPSCs), with no changes in their amplitudes after CSDS (**Figure 5A-C**). Notably, the frequencies and amplitudes of mEPSCs in pyramidal neurons of *P2rx2*-cKO mice were kept intact after CSDS (**Figure 5A-C**). Next, we recorded the pyramidal neurons of mPFC in OE-P2X2 or control mice. Overexpression of P2X2 was sufficient to decrease the frequencies of mEPSCs, and with no changes in their amplitudes (**Figure 5D-F**). These results indicated that P2X2 regulated excitatory synaptic transmission in social stress paradigm.

### Loss- or gain-of-function of P2X2 at pyramidal neurons leads to diametrically opposite changes in dendritic arborization and spine density

**Figure 6:**
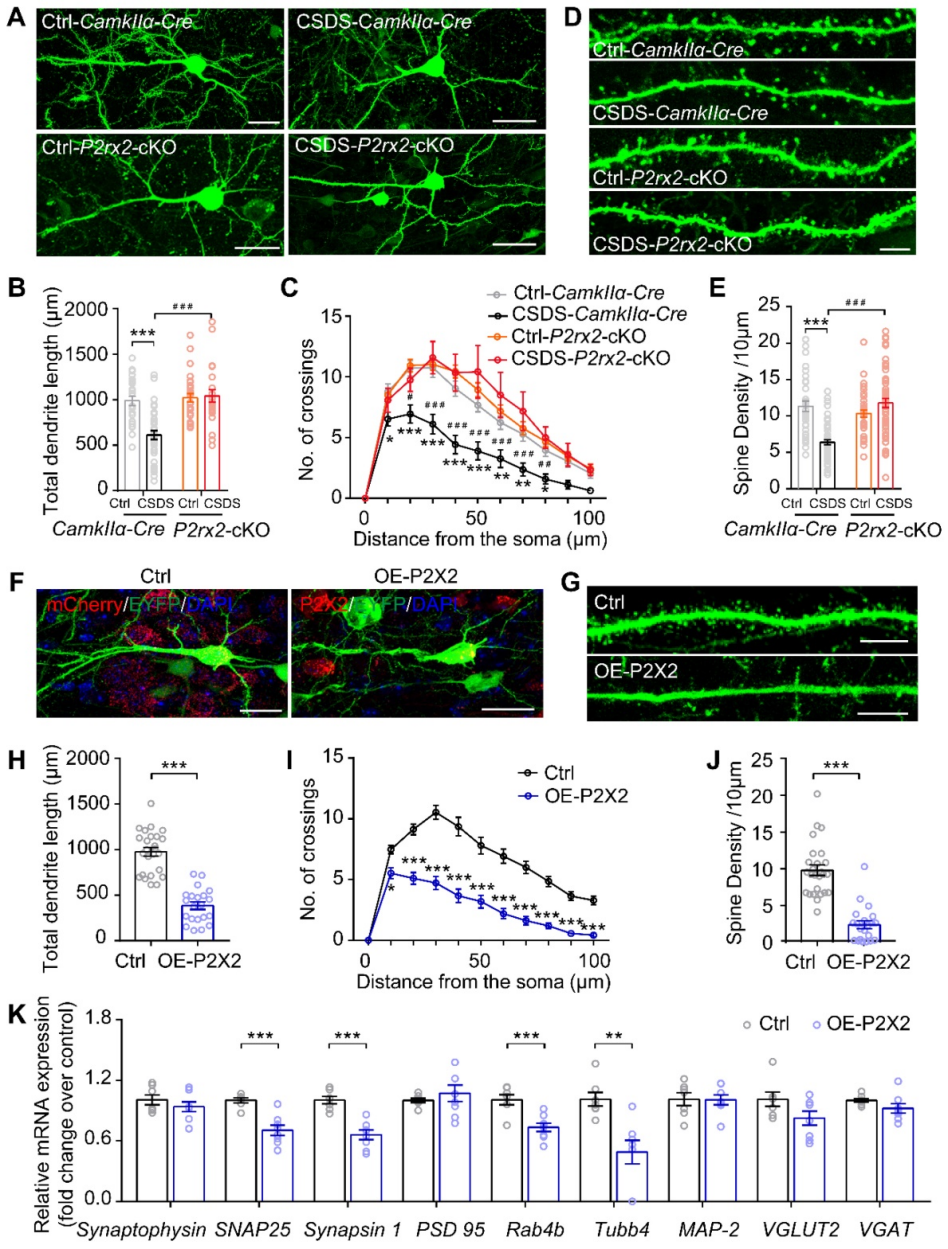
** Loss- or gain-of-function of P2X2 at pyramidal neurons leads to diametrically opposite changes in dendrite branching and spine density.** (**A-C**) Representative confocal images of pyramidal neurons infected with AAV-fDIO-EGFP and AAV-FLP (A), quantification of total dendrite length (B) (n = 23-35 neurons from 3-4 mice, F_(1, 108)_ = 13.453, p < 0.001, two-way ANOVA) and Sholl analysis (C) (n = 23-35 neurons from 3-4 mice, F_(30, 770)_ = 3.531, p < 0.001, two-way ANOVA) in the mPFC of *P2rx2*-cKO or control mice after CSDS paradigm. Scale bar, 10 µm, * compared with ctrl-*CamkIIα-Cre* mice, ^#^ compared with CSDS-*CamkIIα-Cre* mice. (**D-E**) Representative images of dendritic segments (D) (Scale bar, 5 µm), quantification of total spine density (E) (n = 36-58 neurons from 3-4 mice, F_(1, 179)_ = 34.185, p < 0.001, two-way ANOVA, ***p < 0.001 compared with ctrl - *CamkIIα-Cre* mice, ^###^p < 0.001 compared with CSDS - *CamkIIα-Cre* mice) of the pyramidal neurons of *P2rx2*-cKO or control mice after CSDS paradigm. (**F-G**) Representative pictures of pyramidal neurons (F) (Scale bar, 10 µm) and dendritic segments (G) (Scale bar, 5 µm) in the mPFC of OE-P2X2 or control mice. (**H-J**) Statistical comparison of total dendrite length (H) (t_(44)_ = 9.310, *p* < 0.001, unpaired *t* test), Sholl analysis (I) (F_(10, 440)_ = 11.553, p < 0.001, two-way ANOVA) and total spine density (J) (t_(44)_ = 7.960, *p* < 0.001, unpaired *t* test) in the OE-P2X2 or control neurons (n = 21-25 neurons from 3 mice/group). (**K**) mRNA levels of gene profiles related to synaptic-related genes in the mPFC of OE mice and their littermate control (n = 7-8, one-way ANOVA). The data are shown as mean ± SEM. *p < 0.05, **p < 0.01, ***p < 0.001; ^#^p < 0.05, ^##^p < 0.01, ^###^p < 0.001.

Chronic stress causes dendritic atrophy and spine loss, which leads to dysfunction of neurons 41, 42, 47. To further investigate the neuronal morphological change in *P2rx2*-cKO and control mice in response to chronic stress, we examined the dendritic arborization and spine density in mPFC pyramidal neurons. To do this, we sparsely labeled *CamkIIα* positive neurons by injecting AAV-DIO-Flp and AAV-fDIO-EYFP, an AAV harboring a double-FRT-flanked inverted open reading frame (fDIO) that expressed EYFP in an Flp-dependent manner, in the mPFC of *P2rx2*-cKO and control mice (**Figure 6A**). Morphometric analysis revealed that pyramidal neurons in control mice showed a significant decrease in the total dendritic length (**Figure 6B**), dendrite complexity (**Figure 6C**) and spine density after CSDS experiments (**Figure 6D-E**). In contrast, the total dendritic length (**Figure 6B**), dendrite complexity (**Figure 6C**) and spine density (**Figure 6D-E**) in *P2rx2*-cKO mice were still intact after CSDS.

Next, we morphometrically analyzed the pyramidal neurons of OE-P2X2 or control mice injected with AAV-DIO-Flp and AAV-fDIO-EYFP virus, simultaneously (**Figure 6F**). Overexpression of P2X2 was sufficient to decreased total dendritic length (**Figure 6G**), complexity of dendrites (**Figure 6H**) and spine density (**Figure 6I-J**) in the mPFC of OE-P2X2 mice compared with littermate control. Furthermore, RT-qPCR analysis demonstrated significant decreases for 4 of the 8 synapse-related genes (*SNAP 25*, *Synapsin 1*,* Rab4b*, *Tubb4*) in the mPFC of OE-P2X2 mice compared with littermate control (**Figure 6K**).

Taken together, these data demonstrated that conditional knockout or overexpression of P2X2 in pyramidal neurons induced diametrically opposite changes in dendritic arborization and spine density.

## Discussion

Overall, we found that chronic stress induced an upregulation of P2X2 levels in the mPFC, and P2X2 bidirectionally mediated stress vulnerability by regulations of neuroplasticity. Our studies established a new P2XRs pathway for behavioral homeostatic adaptation, and the extent of P2X2 function in emotion regulation.

Postmortem studies of MDD subjects reported that a reduction in the density of neurons and glial cells 48-50, and decrease the number of synapses in the PFC 40, 51. Rodent studies have provided detailed evidence of astrocytic dysfunction, neuronal atrophy, reduced synaptic density, and disruption of synaptic plasticity in PFC in models of depression 41, 42, 52, 53, indicating that maladaptive neuron-glia interactions play a key role in pathophysiology of depression. Our previous study reported that astrocytic ATP mediates depressive-like behaviors 15. Besides, P2X2 receptors which are activated by ATP, are the predominant P2X receptors expressed on neurons 17, 54-56. Here, our findings showed that P2X2 levels in mPFC are significantly increased in depressive susceptible mice after CSDS paradigm (**Figure 1**), P2X2-cKO mice display resilience to social stress-induced depressive-like behavior, whereas OE-P2X2 mice increase susceptibility to stress (**Figure 2, 3**). These results reveal a critical role of P2X2 in mood disorder, and ATP-P2X2 signaling pathway bridges the dysfunction of astrocytes and neurons in the PFC.

Previous studies and our results have revealed that depression induces a hypoactive state characterized of mPFC pyramidal neurons through a decrease in glutamatergic synaptic input 24, 57, 58 ( **Figure 5**), an increase in GABAergic synaptic input 59, 60 and a reduction in the neuronal excitability 58 (**Figure 4**). Although P2X2 abundantly express on *CamkIIα*-positive excitatory neurons (**Figure 1**), it also express on the GABAergic neurons in mPFC 60, 61**.** Nevertheless, previous studies also suggested that ATP regulates glutamatergic neurons transmission 62-64 and inhibitory synaptic transmission in brain 61, 65. Our group and others' studies have shown that reduction in the level of extracellular ATP levels in the mPFC is implicated in depression induced by CSDS 15, 24, 64, 66. GABAergic interneuron has higher spikes frequency than pyramidal neurons when activated 57, 67, thus may be more effectively response to the reduced ATP level and contribute to antidepressant-like effect in FST and SPT 60. Meanwhile, P2X2 was specifically increased in the mPFC after 10 days CSDS paradigm (**Figure 1**); P2X2 protein and mRNA levels were decreased chronic treated with ATPᵞS, or increased after ATPase treatment in cultured neurons (**Figure 1**), indicating that the P2X2 levels may be dynamic slowly response to ATP concentration. Previous studies and our results showed that *P2rx2^-/-^, P2rx2*-cKO mice and OE-P2X2 mice displayed no obvious difference in FST 68, 69 (**Figure 2H, Figure 3F**). Intriguingly, in SPT and CSDS, *P2rx2*-cKO mice showed antidepressant-like behavior (**Figure 2E-G**), while OE-P2X2 mice produced depressive-like behaviors (**Figure 3D-E**). These results suggested P2X2 in pyramidal neurons may play chronic response to the ATP levels and contribute to antidepressant-like effect in SPT and CSDS. However, the intracellular mechanism of P2X2 in depression needs to be further studied, and it is worth to examine the coordination of glutamatergic synaptic input and GABAergic synaptic input on mPFC pyramidal neurons in depression in future study.

Activity-dependent remodeling of synaptic AMPARs depends on the elevation of intracellular Ca^2+^, and CaMKII, the most notable Ca^2+^-dependent protein kinases, is required for remodeling of synaptic AMPARs 70, 71. Electron microscopy images showed that P2X2 receptors expressed on pyramidal neurons that appear to be highly mobile at the plasma membrane but remain at the edge of glutamatergic synapses 39. And activation of postsynaptic P2X2 decreases AMPAR trafficking and synaptic strength at glutamatergic synapses 17. Besides, chronic stress leads to reduction in synaptic strength 72, 73. The number of synapses is reflected in the frequency of mEPSCs. Consequently, the size and number of mEPSCs are closely correlated with the physical size of synapses and number of spines 74, 75. The spine density increases with enhanced synaptic activity, and shrinks or is replaced by filopodia when synaptic activity is low 74. These studies suggested that P2XRs may regulate synaptic morphology and function via AMPARs trafficking. Furthermore, our result showed that Patch-clamp recording and morphological analysis found that P2X2 receptors contributed to stress-induced alterations of synapse transmission and dendritic morphology (**Figure 5-6**). and synaptic-related genes were down regulated in P2X2 overexpression mice (**Figure 6K**), suggesting P2X2 may directly regulated dendritic morphology and synapse function though mediating the expression of synaptic-related genes. However, the detail mechanism needed to be further studied.

Together, our study defines that P2X2 in pyramidal neurons is sufficient and necessary to regulate neuronal plasticity in mPFC and depressive-like behaviors. These findings identify the regulation of excitatory synapses onto CamkIIα mPFC neurons by P2X2 as an important mechanism underlying mood disorder, representing a potential medicinal target for major depressive disorder.

## Supplementary Material

Supplementary figures and table.

## Abbreviations

P2X2: purinergic subtype X2 receptors
ATP: adenosine triphosphate
mPFC: medial prefrontal cortex
CSDS: chronic social defeat stress model
MDD: major depressive disorder
PTSD: posttraumatic stress disorder
RT-qPCR: real-time quantitative polymerase chain reaction
RNA: Ribonucleic Acid
cDNA: complementary deoxyribonucleic acid
PVDF: polyvinylidene fluoride
GAPDH: glyceraldehyde-3-phosphate dehydrogenase
OCT: opti-mum cutting temperature compound
PBS: phosphate-buffered saline
ATPᵞS: adenosine triphosphate gamma S
ATPase: adenosine triphosphatase
SSDS: subthreshold social defeat stress
SI: social interaction
FIT: forced interaction test
FST: forced swimming test
NSF: novel stressed feeding
OFT: open field test
ANOVA: analysis of variance
cKO: conditional knockout
OE: overexpression
mEPSCs: miniature excitatory postsynaptic currents
GCaMP: GFP-calmodulin-M13 peptide
EYFP: enhanced yellow fluorescent protein
EGFP: enhanced green fluorescent protein
AMPARs: α-amino-3-hydroxy-5-methyl-4-isoxazole-propionicacid receptors
CaMKII: Ca^2+^-dependent protein kinases II
