# Supplementary material for: P2X2 receptors in pyramidal neurons are critical for regulating vulnerability to chronic stress
Source: Theranostics. 2022 May 1;12(8):3703–18. doi: 10.7150/thno.72144 (PMC9131261; doi:10.7150/thno.72144)
Supplement: Supplementary file 1 — Supplementary figures and table. [file thnov12p3703s1.pdf]

Supplementary Information

P2X2 receptors in pyramidal neurons are critical for regulating vulnerability to chronic stress

Xiao-Jing Kuang[1,#], Can-Yuan Zhang[1,#], Bing-Yi Yan[1], Wei-Zhong Cai[1], Cheng-Lin Lu[1], Li-Jia Xie[1], Shu-Ji Li[1], Peng-Li Kong[1], Jun Fan[1], Shu-Min Pan[1], Ting Guo[1] and Xiong Cao.[1,2 *]

1. Key Laboratory of Mental Health of the Ministry of Education, Guangdong-Hong Kong-Macao Greater Bay Area Center for Brain Science and Brain-Inspired Intelligence, Guangdong Province Key Laboratory of Psychiatric Disorders, Department of Neurobiology, School of Basic Medical Sciences, Southern Medical University, Guangzhou 510515, P. R. China.

2. Microbiome Medicine Center, Department of Laboratory Medicine, Zhujiang Hospital, Southern Medical University, Guangzhou, Guangdong 510515, P. R. China.

# These authors contributed equally to this work.

* Correspondence should be addressed to caoxiong@smu.edu.cn

Figure. S1

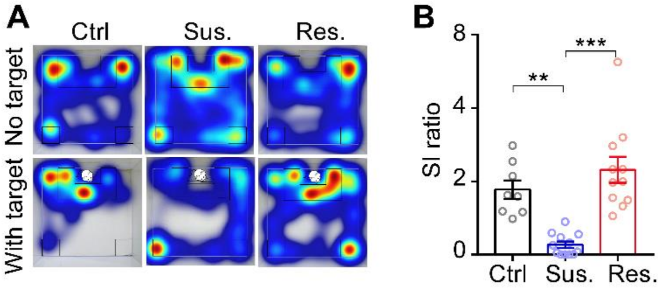

Figure. S1. Social interaction test. (**A**) Representative heatmaps of normalized time spent during SI test of C57BL/6J mice. (**B**) Statistical comparison of SI ratio after CSDS (n = 8 -12, n = 8 -12, *p = 0.042, one-way ANOVA). The data are shown as mean ± SEM. *p < 0.05, **p < 0.01, ***p < 0.001.

Related to Figure 1.

Figure. S2.

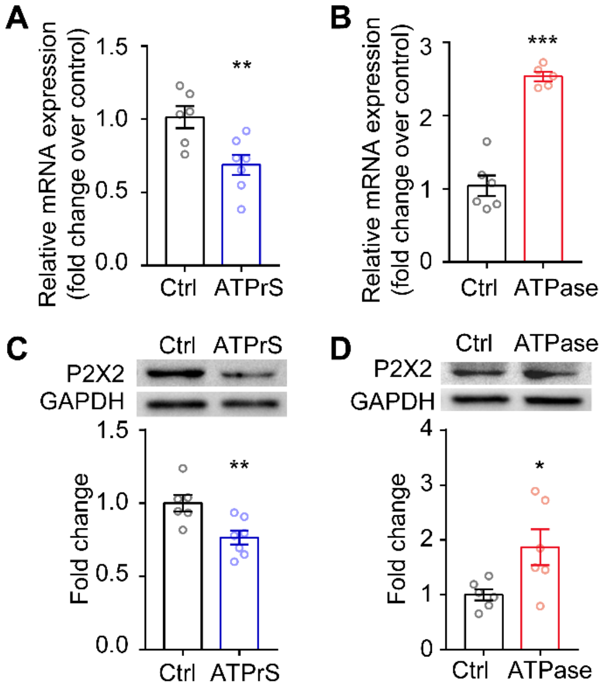

Figure. S2. P2X2 expression of cultured PFC neurons treated with ATPγS or ATPase treatment. (**A-B**) Statistic analysis of P2X2 mRNA expression in the PFC cultured neurons after ATPγS (A) ($n = 6\text{-}7$, $t_{(11)} = 3.203$, $p = 0.008$, unpaired t test) or ATPase treatment (B) ($n = 5\text{-}6$, $t_{(9)} = 8.968$, $p < 0.001$, unpaired t test). (**C-D**) Western blots representation (top) and quantification (bottom) of P2X2 protein level in the PFC cultured neurons after ATPγS (C) ($n = 6\text{-}7$, $t_{(11)} = 3.734$, $p = 0.003$, unpaired t test) or ATPase treatment (D) ($n = 6$, $t_{(10)} = 2.520$, $p = 0.030$, unpaired t test). The data are shown as mean ± SEM. *$p < 0.05$, **$p < 0.01$, ***$p < 0.001$.

Figure. S3

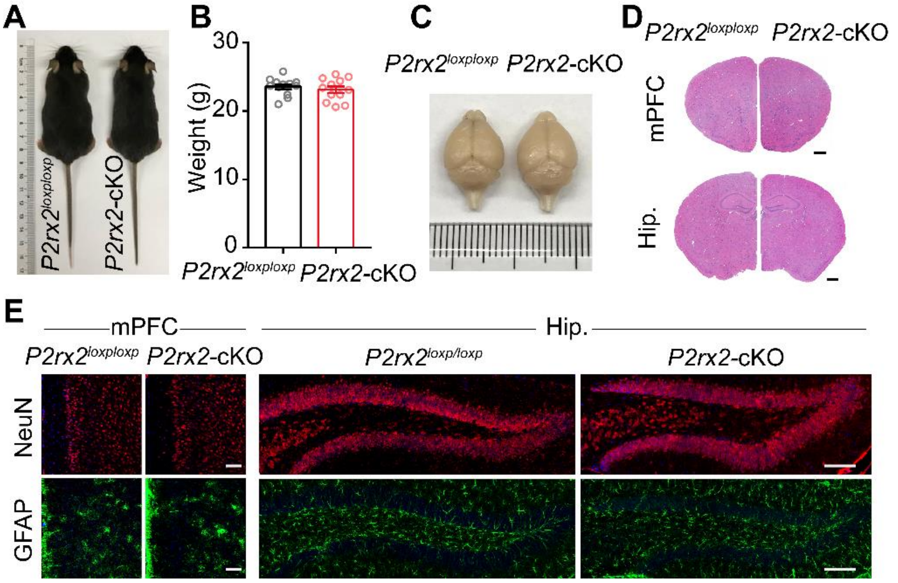

Figure. S3 Generation of *P2rx2*-cKO mice. (**A-B**) Representative images (A) and body weight (B) (n = 12, $t_{(22)} = 0.209$, $p = 0.840$; unpaired t test) of adult *P2rx2*-cKO and control mice. (**C-D**) Gross appearance (C) and H&E-stained coronal sections (mPFC or hippocampus, Scale bar, 500 µm) of the brain (D) of *P2rx2*-cKO or littermate control mice. (**E**) Immunofluorescence for NeuN (red) and GFAP (green) in the mPFC or hippocampus of *P2rx2*-cKO and littermate control mice. Scale bar, 50 µm. The data are shown as mean ± SEM.

Related to Figure. 2.

Figure. S4

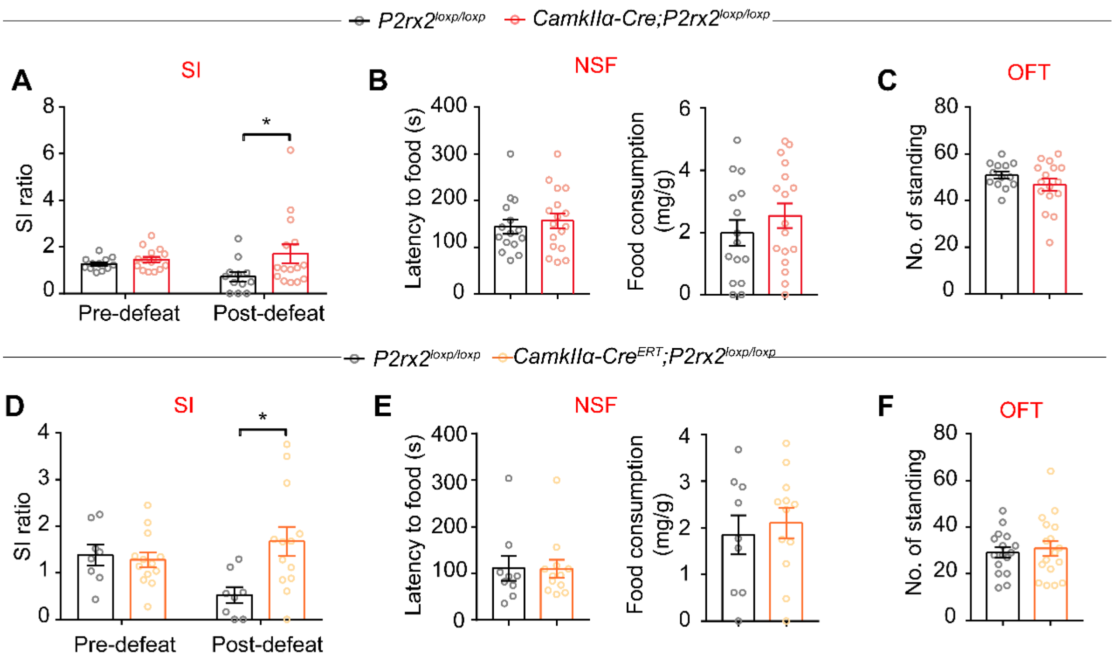

Figure. S4. Relative behaviors of *P2rx2* conditional knockout mice. (**A**) SI ratio before and after CSDS of *P2rx2*-cKO and control mice (n = 12-14, p = 0.042, interaction effect, matching two-way ANOVA). (**B-C**) Statistics analysis of *P2rx2*-cKO and control mice in NSF (B, latency to food: $t_{(30)} = 0.579$, p = 0.570; food consumption: $t_{(30)} = 0.960$, p = 0.340; n = 15-17, unpaired t test) and OFT (C, number of standing: n = 13-16, $t_{(27)} = 1.288$, p = 0.210, unpaired t test). (**D**) Social interaction ratio before and after CSDS of *CamkIIα-Cre*^{ERT}; *P2rx2*$^{loxp/loxp}$ and control mice (n = 8-13, p = 0.030, interaction effect, matching two-way ANOVA). (**E-F**) Statistics analysis of *CamkIIα-Cre*^{ERT}; *P2rx2*$^{loxp/loxp}$ and control mice in NSF (E) (latency to food: $t_{(19)} = 0.018$, p = 0.986; food consumption: $t_{(19)} = 0.491$, p = 0.629; n = 9-12, unpaired t test) and OFT (F) (number of standing: n = 14-16, $t_{(31)} = 0.421$, p = 0.680, unpaired t test). The data are shown as mean ± SEM. *p < 0.05.

Related to Figure 2.

Figure. S5

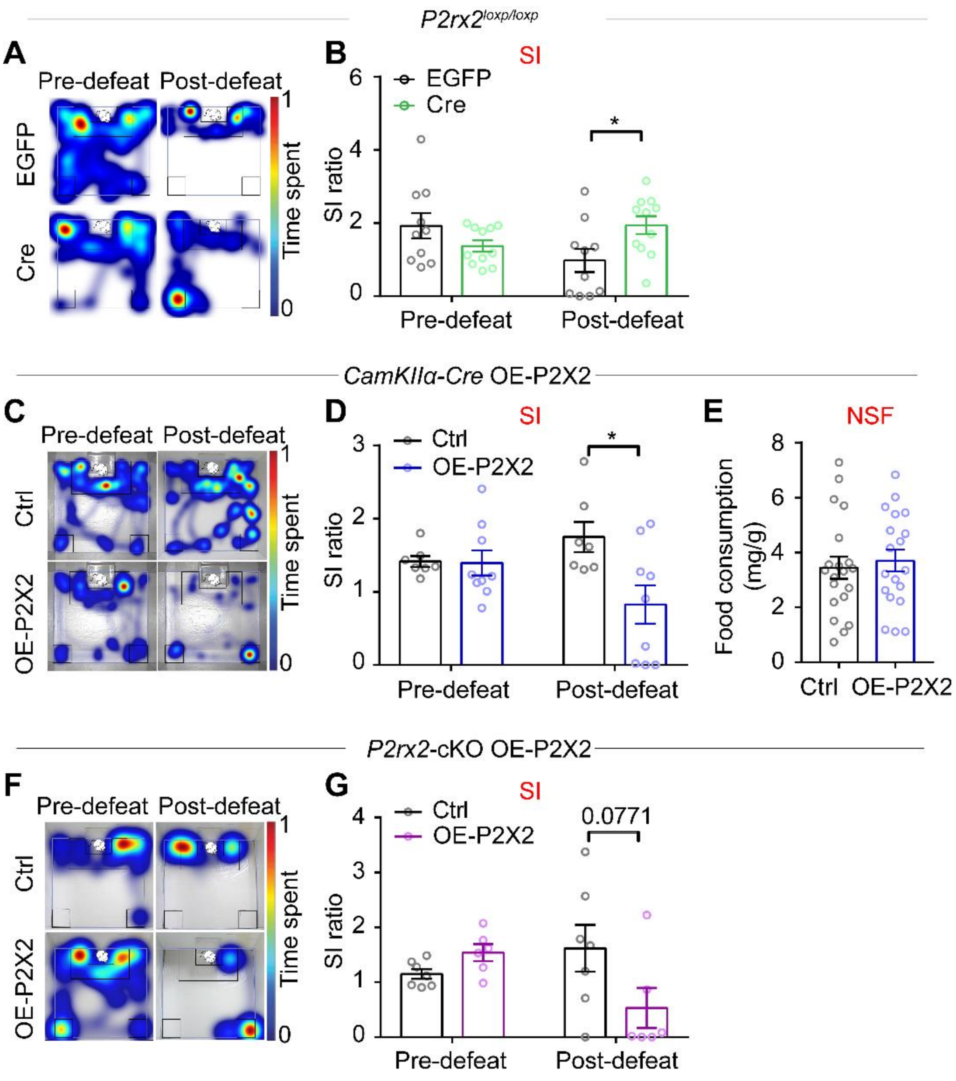

Figure. S5. Relative behaviors of P2X2 local knockdown or overexpression mice. (**A**) Representative heatmaps of normalized time spent during SI test before (left) and after (right) CSDS of *P2rx2*$^{loxp/loxp}$ mice injected with AAV-CamkIIα-EGFP-Cre or control virus. (**B**) SI ratio (n = 10-11, p = 0.010, interaction effect, matching two-way ANOVA) of *P2rx2*$^{loxp/loxp}$ mice injected with AAV-CamkIIα-EGFP-Cre or control virus. (**C**) Representative heatmaps of normalized time spent during SI test before (left) and after (right) CSDS of *CamkIIα-Cre* mice injected with AAV-DIO-P2X2 or control virus. (**D-E**) SI ratio (D) (n = 7-9, p = 0.035, interaction effect, matching two-way ANOVA) and NSF (E) (n = 11-12, $t_{(21)} = 0.384$, p = 0.700; unpaired t

test) of *CamkIIα-Cre* mice injected with AAV-DIO-P2X2 or control virus. (**F**) Representative heatmaps of normalized time spent during SI test before (left) and after (right) CSDS of *P2rx2*-cKO mice injected with AAV- DIO-P2X2 or control virus. (**G**) SI ratio ($n = 6\text{-}7$, $p = 0.025$, interaction effect, matching two-way ANOVA) of *P2rx2*-cKO mice injected with AAV- DIO-P2X2 or control virus. The data are shown as mean ± SEM. *$p < 0.05$.

Related to Fig. 3.

Figure. S6

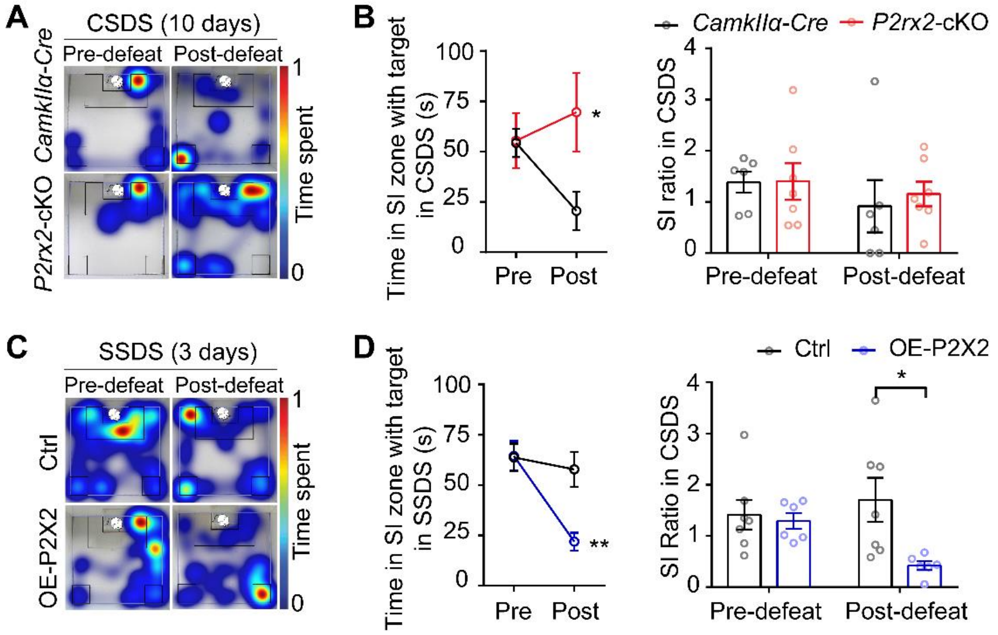

Figure. S6. CSDS results of mice applying AAV-DIO-GCaMP6s virus. (**A, B**) Representative heatmaps (A) and analysis of social interaction (B) (n = 6-7, time in the interaction zone with target: p = 0.017; SI ratio: p = 0.747; interaction effect, matching two-way ANOVA) of *P2rx2*-cKO and *CamkIIα-cre* control mice injected with AAV-DIO-GCaMP6s virus in CSDS paradigm. (**C, D**) Representative heatmaps (C) and analysis of social interaction (D) (n = 6-7, time in the interaction zone with target: *p = 0.035; SI ratio: p = 0.060; interaction effect, matching two-way ANOVA) of P2X2 overexpression and control mice injected with AAV-DIO-GCaMP6s virus. The data are shown as mean ± SEM. *p < 0.05, **p < 0.01.

Related to Figure 4.

Supplementary Table 1

Gene	Former primer	Reverse primer
P2rx1	CCG AAG CCT TGC TGA GAA	GGT TTG CAG TGC CGT ACA T
P2rx2	CAG AAC TGG CAC ACA AGG G	CAG TCA CAC AGA AAG GAG CC
P2rx3	GGT GGC TGC CTT CAC TTC	TCA GCC CCT TTG AGG AAA
P2rx4	CCA ACA CTT CTC AGC TTG GAT	TGG TCA TGA TGA AGA GGG AGT
P2rx5	CAC AGT CAT CAA CAT TGG TTC C	AGG TAG ATA AGT ACC AGG TCA CAG AAG
P2rx6	TGT CCC CAG TAC TCC TTC CA	CAC CAG TGA TTG GCT GTC C
P2rx7	GGG GGT TTA CCC CTA CTG TAA	GCT CGT CGA CAA AGG ACA C
Synaptophysin	TCT TTG TCA CCG TGG CTG TGT T	TCC CTC AGT TCC TTG CAT GTG T
SNAP 25	CTG GCA TCA GGA CTT TGG TT	ATT ATT GCC CCA GGC TTT TT
Synasin 1	CCA GCT CAA CAA ATC CCA GT	GGT GTC AGT CGG AGA AGA GG
PSD 95	TCT GTG CGA GAG GTA GCA GA	AAG CAC TCC GTG AAC TCC TG
Rab4b	ACT ATT GGC GTG GAG TTT GG	CAC AGA GGA TGA CCA CGA TG
Tubb4	GGG GAC CTC AAC CAC CTA GT	ATC CTG GCA TGA AGA AGT GG
MAP 2	TCA GGA GAC AGG GAG GAG AA	GTG TGG AGG TGC CAC TTT TT
VGLUT2	GCT CAC CTC TAC CCT CAA TAT	CCA CTT GCT CCA TAT CCC ATG

	G	
VGAT	ACG ACA AAC CCA AGA TCA CG	AAG ATG ATG AGG AAC AAC CCC
β-actin	CCA CCA TGT ACC CAG GCA TT	CGG ACT CAT CGT ACT CCT GC

Supplementary Table 1**. qPCR primers.**
